# Outcomes and Complications Associated with Mechanical Thrombectomy in the Treatment of Acute Ischemic Stroke

**Published:** 2024-12-10

**Authors:** Zubair Ahmed, Jeremy Pan, Tony Eskandar, Devendra K. Agrawal

**Affiliations:** Department of Translational Research, College of Osteopathic Medicine of the Pacific, Western University of Health Sciences, Pomona CA 91766, USA

**Keywords:** Acute ischemic stroke, Brain ischemia, Endovascular therapy, Intravenous thrombolysis therapy, Mechanical thrombectomy, Stroke

## Abstract

Universally, stroke presents as neurological deficits due to the obstruction of blood supply to specific regions of the brain. Among the three main categories of stroke, acute ischemic stroke is the leading cause of death and disability worldwide. As of today, there are two effective treatment methods: thrombolysis and endovascular therapy. Intravenous thrombolysis treatment via tissue plasminogen activator is typically administered within 4.5 hours from the onset of symptoms. Mechanical thrombectomy, a type of endovascular therapy, is indicated for acute ischemic stroke due to a large vessel occlusion occurring within 24 hours since the patient was last seen asymptomatic. Due to the disadvantages of intravenous thrombolysis treatment, such as a limited time window and numerous contraindications, studies have proven the efficacy of mechanical thrombectomy as the standard of care for acute ischemic stroke due to large vessel occlusion in the anterior circulation. Endovascular therapy was associated with higher rates of independent clinical outcome and successful reperfusion rates compared to intravenous thrombolysis treatment. Currently, stent retrievers and aspiration devices are the two most common endovascular therapy techniques. Two prominent studies compared the reperfusion rates between these two techniques, but neither was found to be more beneficial than the other. The decision to use either a stent retriever or direct aspiration depends on the patient and the thrombus involved. This comprehensive article critically discusses the findings on the efficacy of mechanical thrombectomy therapy for acute ischemic stroke and its associated outcomes and complications.

## 1. Introduction

**Figure 1:**
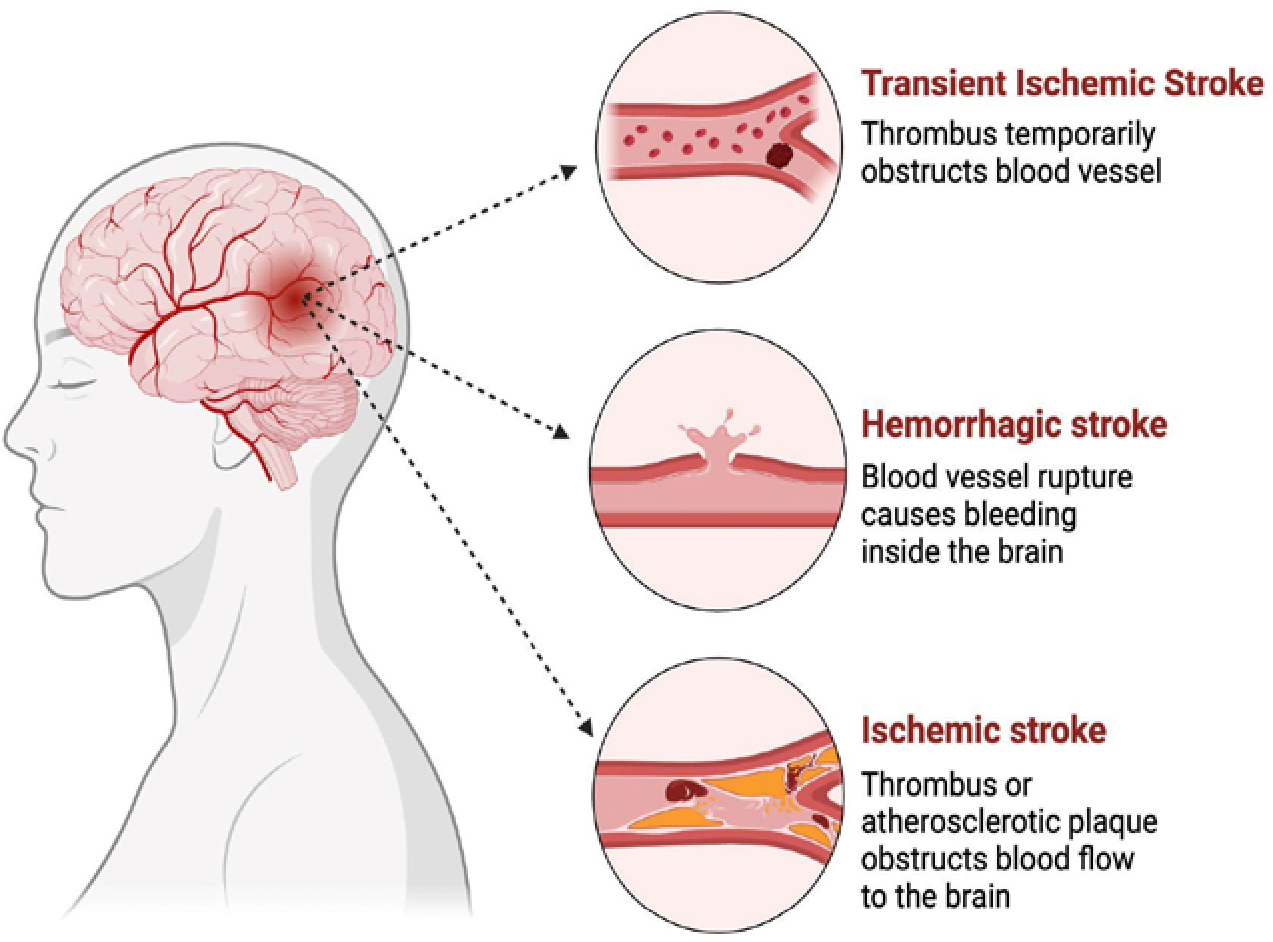
Classification of stroke: Stroke is typically grouped into three main categories: transient ischemic attack, hemorrhagic, and ischemic. The causes of each type of stroke could be different with varying underlying pathophysiology, leading to resistance to blood flow in brain arteries or blood vessel rupture resulting in brain hemorrhage.

Globally, stroke has been commonly characterized as a neurological impairment resulting from an acute focal injury to the central nervous system because of vascular causes. However, this traditional definition is primarily clinical and fails to highlight the recent scientific developments that have been made toward various attempts to better understand the outcomes of stroke [1]. Recently, the American Heart Association presented a more modern definition of stroke that includes both tissue and clinical criteria. This definition is all-encompassing as it incorporates permanent damage to the brain, spinal cord, or retina due to vascular dysfunction based on clinical presentation, imaging, or pathological evidence, with or without symptoms [1]. Strokes are typically categorized into three main groups: transient ischemic attack (TIA), ischemic stroke, and hemorrhagic stroke (Figure 1). A TIA is a transient blockage of blood flow to a specific region of the brain that leads to neurological dysfunction, typically lasting less than a few minutes and resolving on its own [2]. An ischemic stroke occurs due to obstruction of blood flow to the brain, causing insufficient oxygen delivery [3]. A hemorrhagic stroke takes place when a blood vessel in the brain ruptures, causing blood to pool in the surrounding brain parenchyma [4]. Overall, studies have shown that hemorrhagic stroke accounts for about 13% of all stroke cases, and ischemic stroke accounts for about 87% [5].

When considering progression and impact, ischemic stroke can be grouped into two stages: acute and chronic; however, it is primarily categorized as acute [6]. Acute ischemic stroke is the leading cause of death and disability globally and is classified as vascular changes that occur within the first 24 hours since onset [6,7]. When there is a lack of oxygen due to a blood clot in the brain, an acute ischemic stroke can present as a sudden onset of weakness or numbness to one side of the body, confusion, vision changes, difficulty speaking, and loss of balance and coordination. The BEFAST (Balance, Eyes, Face, Arm, Speech, Time) acronym is a well-renowned tool used by millions of people across the world to assist them in recognizing stroke symptoms and how to act accordingly [8]. Although there is some variability in time intervals, the chronic stage of ischemic stroke is typically categorized as vascular etiologies that present 3–6 months after onset [6]. Long-term symptoms include speech impairments, restricted physical abilities, weakness to one side of the body, and difficulty gripping and holding objects [9]. Additionally, rehabilitation programs are available that can help improve these chronic effects, such as speech, physical, and cognitive therapy.

There are only two proven effective treatment options for acute ischemic stroke: intravenous thrombolysis therapy and endovascular therapy [10]. The intravascular thrombolysis therapy is a thrombolytic therapy that involves the administration of a medication called tissue plasminogen activator (tPA) within 3 hours and no longer than 4.5 hours [12,13]. This drug dissolves blood clots which can help restore blood flow back to the brain and reduce the risk of neurological dysfunction and organ damage. Mechanical thrombectomy, a type of endovascular therapy, is a minimally invasive procedure that involves the usage of a catheter to remove the blood clot under imaging guidance [14,15]. Mechanical thrombectomy is indicated for acute ischemic stroke due to a large vessel occlusion occurring within 24 hours since the last time the patient was seen asymptomatic [13]. In a clinical trial of 136 patients, “Thrombolysis With Alteplase at 0.6 mg/kg for Stroke With Unknown Time of Onset (THAWS)”, 36% patients were diagnosed with cardioembolic stroke and were treated with MRI-guided intravenous thrombolysis. There was no incidence of symptomatic intracranial hemorrhage or parenchymal hematoma Type II following thrombolysis [16]. These findings support the successful use of MRI-guided intravenous thrombolysis in patients with cardioembolic stroke with unknown time onset.

## 2. Drawbacks and Efficacy

Acute ischemic stroke has been a significant contributor to worldwide causes of mortality and disability. Although intravascular thrombolysis therapy treatment has been more commonly used over the last couple of decades, there are major drawbacks that limit its efficacy. First, intravascular thrombolysis therapy must be delivered within 4.5 hours from the onset of symptoms. In the 2009 European Cooperative Acute Stroke Study III (ECASS III), the effectiveness of tPA administration from 3 hours to 4.5 hours was evaluated compared to the original 0–3 hours’ time window [17]. It was found that intravascular thrombolysis therapy treatment 3–4.5 hours after the appearance of stroke-like symptoms had a significantly better outcome at 90 days compared to control and there was no increased risk of intracranial hemorrhage or mortality compared to the 0–3 hours’ time window [17]. If tPA was administered after 4.5 hours and up to 6 hours after stroke onset, studies have shown that there is some improvement in functional outcomes, but it is not statistically significant. Additionally, there was an increased risk of intracranial hemorrhage [18,19]. However, some studies have shown that intravenous tPA delivery after 4.5 hours and up to 12 hours was safe and effective, but this time marker has yet to be used in clinical practice [20,21]. Another disadvantage of intravascular thrombolysis therapy is the wide variety of contraindications such as recent surgery, active bleeding, and coagulation abnormalities [22]. Furthermore, admission time delays have been a prominent factor that excludes patients from thrombolysis therapy given the time-sensitive nature of this treatment [23].

Due to these disadvantages of intravascular thrombolysis therapy and the innovative technological advancements that have been made with endovascular therapy, mechanical thrombectomy has recently been established as the gold standard of care for acute ischemic stroke with large vessel occlusion in anterior circulation [24]. Mechanical thrombectomy involves the utilization of a catheter to remove the blood clot. The surgeon begins the procedure by making a small incision on the patient’s wrist, giving them access to an artery. Afterward, they insert the catheter along with the stent retriever and guide it to the site of the obstruction. The stent retriever is used to push through the clot and expand the arterial wall, which allows it to capture the clot. Then, the surgeon can remove the entire clot by pulling out the catheter [25]. According to the American Heart Association (AHA), this technique is most effective when given less than six hours from the onset of stroke [26]. However, based on the recent DAWN1 and DEFUSE3 trials, it was found that mechanical thrombectomy can be beneficial between 6–16 hours and 6–24 hours after the onset of stroke symptoms, respectively [27]. An additional study found that by increasing the time window from 6–24 hours, there was a 26.7% increase in the number of patients receiving thrombectomy for stroke treatment which resulted in a 36.4% increase in independent clinical outcome [28]. Thus, new guidelines state that mechanical thrombectomy can be used up to 24 hours after stroke.

Numerous studies have investigated the efficacy of mechanical thrombectomy for the treatment of acute ischemic stroke. The first three randomized clinical control trials that compared endovascular therapy to intravascular thrombolysis therapy were the SYNTHESIS Expansion, IMS III, and MR RESCUE, all of which did not find any significant differences in clinical outcomes between the treatments [24]. This was because the studies did not include a stent retriever during mechanical thrombectomy and a lack of specific criteria to select large occluded vessels [24]. The SWIFT PRIME, ESCAPE, REVASCAT, EXTEND-IA, and MR CLEAN are a series of randomized clinical trials that rectified these issues. This resulted in a significant increase in clinical outcomes and recanalization rates when compared to thrombolytic treatment or other therapies [29,30]. Furthermore, another study conducted a meta-analysis of five systematic literature reviews comparing mechanical thrombectomy to intravascular thrombolysis therapy. It was found that for every four to six patients who received mechanical thrombectomy, one more patient will be able to regain independent function at 90 days, compared to the patients who only received thrombolytic treatment alone [31]. Moreover, mechanical thrombectomy not only significantly improves functional outcomes in patients, but it is also more cost-effective compared to thrombolytic treatment [32]. Based on these sets of clinical trials, mechanical thrombectomy became universally accepted as an effective therapeutic technique for the treatment of acute ischemic stroke.

The arrival of the stroke patient to a treatment facility varies. This is because the acute ischemic stroke might have occurred during sleep or due to the late onset symptoms and the time required to seek medical attention. Nonetheless, the length of time between the initial symptoms and the specific treatment is critical for effective and safe treatment [33]. Indeed, MRI could identify patients who had symptom onset within last 4.5 hours for the thrombolytic therapy. Currently, there is an ongoing WAKE-UP clinical trial which is a European, multicenter, randomized, double-blind, placebo-controlled in patients with unknown time of symptom onset and treated with recombinant tPA or placebo [34]. The findings of this trial will be useful for many acute ischemic stroke patients who are currently excluded from specific acute therapy.

The effects of intravascular thrombolysis therapy administration before applying mechanical thrombectomy (bridging therapy) versus mechanical thrombectomy treatment alone for acute ischemic stroke due to large vessel occlusions in anterior circulation have also been thoroughly examined; however, there have been differing results. Some studies found no significant differences in the recanalization rates and adverse effects between mechanical thrombectomy alone and bridging therapy [35]. Similarly, another study found no significant differences in the rate of reperfusion and procedural complications between the two treatment modalities [36,37]. On the other hand, a recent study has demonstrated that the combination of mechanical thrombectomy and intravascular thrombolysis therapy was associated with better rates of survival, increased rates of reperfusion, and more successful recanalization compared to mechanical thrombectomy alone [38,39]. Additionally, there is increased functional independence and successful reperfusion, and lower 90-day mortality when bridging therapy was used compared to solely mechanical thrombectomy [40,41]. Furthermore, the application of bridging therapy had higher rates of minimal disability at the time of discharge from the hospital compared to patients who only received mechanical thrombectomy [42].

## 3. Clinical Outcomes

The clinical or functional outcomes of stroke refer to the possible presence or absence of disabilities that result from a stroke. A good functional outcome means there are no difficulties, and the patient can carry out their daily activities without any symptoms. A poor functional outcome means the patient is experiencing complications after a stroke that may hinder how they live their daily lives. A Modified Rankin Scale is typically utilized to assess the degree of disability in patients who had a stroke in the past and ranges from a scale of 0–6 [43]. A score of 0 represents no symptoms and a score of 6 means that the patient is deceased. In clinical trials, a good functional is designated by a score of 0–2 and a poor functional outcome is a score of 3–6 [44,45].

A recent study gathered data regarding the application of mechanical thrombectomy in the treatment of acute ischemic stroke in the first six years of their practice. Of the 240 patients who received mechanical thrombectomy, good functional outcomes were noted in 50% of the patient population [46]. For the patients who had no neurological impairments before stroke onset, 54% of patients had good functional outcomes [46]. Additionally, symptomatic hemorrhages occurred in 4.6% of patient cases. Furthermore, the association of clinical outcomes was also investigated in pediatric patients with large vessel occlusions. Patients who underwent thrombectomy had much more positive functional outcomes compared to patients who were solely receiving medical management at three months on the pediatric modified Rankin Scale [47]. Moreover, the effects of mechanical thrombectomy on wake-up stroke have also been examined. Wake-up stroke is an ischemic stroke that occurs when a patient is sleeping, with an unknown time of onset as the symptoms are noted by the patient upon waking [48]. These types of strokes account for 20% of all ischemic strokes [49]. One study found that good functional outcomes were present in 46.2 % of patient cases and 83.5% of patients had successful reperfusion [50]. Overall, these results support the fact that mechanical thrombectomy is an effective treatment method that is associated with good functional outcomes.

Like what was seen when comparing bridging therapy to mechanical thrombectomy alone regarding recanalization rates, differing results have also been found when evaluating functional outcomes. A meta-analysis from 2021 found no significant differences were found in functional outcomes at 90 days between bridging therapy and thrombectomy [51]. However, another recent meta-analysis concluded that the combination of thrombolysis and thrombectomy yielded better clinical outcomes than just thrombectomy [52]. These results support the current recommended guidelines for offering thrombolytic treatment to patients who are eligible for mechanical thrombectomy [52].

Additionally, in a cohort study of 2,345 patients, it was found that patients who attained successful recanalization post-thrombectomy were five times more likely to have good functional outcomes compared to individuals who did not achieve successful recanalization [53]. Furthermore, a meta-analysis from 2016 investigated the efficacy of mechanical thrombectomy in improving clinical outcomes. It was shown that mechanical thrombectomy significantly reduced disability at 90 days compared to control patients, especially in patients over 80 years old, those not eligible for thrombolytics, and for patients who have been symptomatic for more than 5 hours [54]. Ultimately, these studies have clearly demonstrated the significant benefit of implementing mechanical thrombectomy in the setting of acute ischemic stroke.

## 4. Safety and Complications

In the United States, stroke accounts for 1 in every 20 deaths and is the main cause of death worldwide [55]. Both thrombolytic and thrombectomy treatments have been proven to be effective treatments for acute ischemic stroke under different circumstances. For mechanical thrombectomy specifically, multiple clinical trials have shown that it is the standard of care for strokes caused by large vessel occlusions [56,57]. However, as with all endovascular procedures, mechanical thrombectomy is associated with significant complications that have been well-documented in many clinical scenarios. These complications can be divided into two main categories: intraprocedural and postprocedural [56]. Intraprocedural risks include issues related to the access site. Postprocedural risks involve risk with recanalization [58]. Overall, both types of complications can be life-threatening and may delay necessary rehabilitation programs. Additionally, these complications increase the length of stay in the hospital, which translates to an increase in the cost as well.

Mechanical thrombectomy procedures are typically performed via the femoral artery using a modified Seldinger technique and then placing 6–8 French sheaths at the site of access [59]. As a result, one of the most common access site complications with the application of mechanical thrombectomy is a groin hematoma [60]. Across a wide array of literature, groin complication rates have shown great variability. Numerous randomized clinical trials have reported a frequency of 2–10% [61–65]. The ESCAPE trial reported a groin hematoma rate of 7.2%. Additionally, The REVASCAT and EXTEND IA trials reported groin hematoma rates of 10.7% and 2.9%, respectively [66]. Retroperitoneal hematoma is another intraprocedural complication that can occur with thrombectomy [67]. The external iliac artery is of retroperitoneal origin and travels below the inguinal ligament to become the extraperitoneal femoral artery [68]. Needle stick puncture wounds above the inguinal ligament can lead to bleeding into the retroperitoneal space due to difficulty in achieving hemostasis. The femoral artery is typically compressed against the femoral head; however, above the inguinal ligament, there is no firm support for the artery, thus making it very difficult to apply enough manual pressure to stop the bleeding [59].

Intracranial hemorrhage is a potentially catastrophic complication that can occur in patients post-thrombectomy [69]. Even though recent technological advances in thrombectomy have resulted in a reperfusion rate greater than 80% in patients with large vessel occlusions, studies have shown that approximately 40% of patients developed intracranial hemorrhage after mechanical thrombectomy [70–72]. A similar study found that among 135 patients who received mechanical thrombectomy for acute ischemic stroke, 38.5% of patients had asymptomatic intracranial hemorrhage and 12.6% and symptomatic intracranial hemorrhage [73]. Serum glucose levels and the number of stent retriever passes were significantly associated with intracranial hemorrhage [73]. Furthermore, another study found that patients with wake-up strokes are more susceptible to symptomatic intracranial hemorrhage, while complete recanalization post-thrombectomy served as a protective measure against intracranial hemorrhage [74]. Additionally, different subtypes of intracranial hemorrhage were present based on the site of occlusion, but the amount of bleeding at each site did not change. Mechanical thrombectomy performed on an occluded middle cerebral artery commonly resulted in a subarachnoid hemorrhage while obstruction to the internal carotid artery often resulted in more severe hemorrhages such as large parenchymal hematomas [72–75].

## 5. Patient Selection

The National Institute of Health Stroke Scale is a quantitative scale used to measure neurological deficits associated with stroke severity [76]. The National Institute of Health Stroke Scale encompasses neurological examination that includes 15 individual elements ranging from motor and sensory function to level of consciousness and attention. All the elements are added together to yield a score ranging from 0–42. This score provides an overall evaluation of the degree of stroke severity [77]. Scores from 1–5, 5–15, 16–20, and greater than 21 represent mild, moderate, moderate to severe, and severe stroke, respectively [78]. The National Institute of Health Stroke Scale has been shown to be very predictive of clinical outcomes after stroke and is heavily used in clinical practice to examine the neurological impairments found in acute ischemic stroke patients to determine the most effective treatments given the patient’s conditions [79]. Additionally, the Alberta Stroke Program Early CT Score (ASPECTS) is an assessment tool used to evaluate ischemic changes in patients with acute ischemic stroke of the anterior circulation, specifically in the middle cerebral artery (MCA) [80]. The ASPECTS considers 10 topographic points, and one point is subtracted from the total score of 10 for each region that shows signs of ischemic damage [81]. Higher ASPECTS have been associated with higher functional outcomes and lesser risk of symptomatic ICH [82].

The series of randomized clinical trials, including SWIFT PRIME, ESCAPE, REVASCAT, EXTEND-IA, and MR CLEAN, including THRACE and PISTE, underscored the efficacy of mechanical thrombectomy as a treatment for acute ischemic stroke. Based on these trials, the American Stroke Association has established guidelines regarding the criteria necessary for the application of mechanical thrombectomy. They recommended mechanical thrombectomy in a functionally independent adult greater than the age of 18 with an NIHSS score and APSECTS of at least 6 [83,84].

Similarly, the standard guidelines of the Society of Neuro Interventional Surgery state that thrombectomy may be used in patients with acute ischemic stroke of the anterior circulation within 6–24 hours since the onset of symptoms and with APSECTS of at least 6 hours [84]. Furthermore, one study found that elderly (age 90 or older) patients have worse outcomes after mechanical thrombectomy, are less likely to attain successful reperfusion, may have a higher risk for hemorrhage, and are less likely to benefit from mechanical thrombectomy compared to younger patients (70 years old) [84].

## 6. Technological Advances

In 2004, the Food and Drug Administration (FDA) approved the first successful clot retrieval device, the Mechanical Embolus Removal in Cerebral Ischemia (MERCI), which attained recanalization in 46% of patients [85]. Prior to the MERCI device, intravenous thrombolytics was the only treatment modality available. Thus, after the development of this novel therapy, the MERCI retriever became the standard of care, especially for patients who could contraindications to thrombolytics. Afterward, the Penumbra system was created in 2008, leading to thrombolysis in grade 2 or 3 myocardial infarction scores in 81.6% of patients [86,87]. Although results were promising, many earlier studies did not find any significant differences between mechanical thrombectomy and standard medical management for treating acute ischemic stroke. It is also important to note that large vessel occlusions, a prominent indicative factor for the need for thrombectomy, were not confirmed in any of these studies [88].

**Figure 2:**
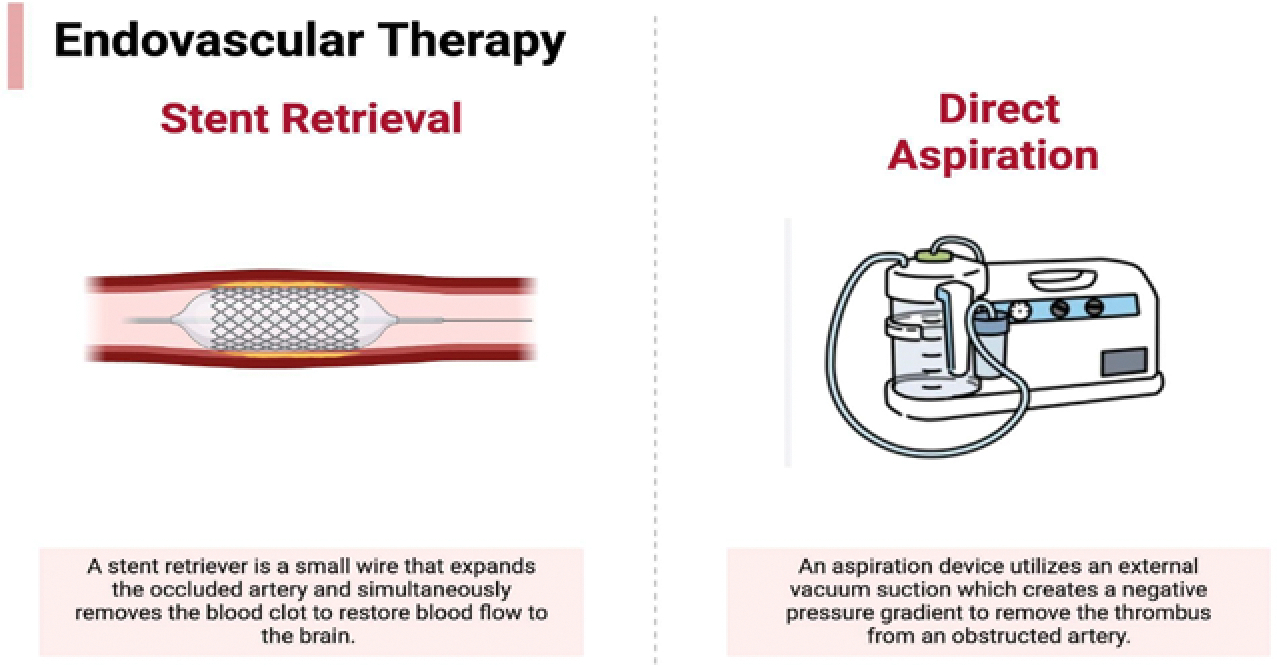
Current strategies of mechanical thrombectomy include stent retrieval and direct aspiration.

The efficacy of endovascular therapy compared to thrombolysis was not demonstrated until after the publication of the SWIFT PRIME, ESCAPE, REVASCAT, EXTEND-IA, and MR CLEAN randomized clinical trials. In 2015, the American Stroke Association updated its treatment guidelines for acute ischemic stroke by including mechanical thrombectomy as the standard of care for large vessel occlusions [89]. Now, two endovascular therapy techniques are commonly used: stent retrievers and aspiration devices (Figure 2). A stent retriever looks like a small metal cage that expands the occluded artery, which reestablishes blood flow, while simultaneously capturing the blood clot and removing it from the patient entirely [90]. Alternatively, aspiration devices utilize an external aspiration pump, creating a negative pressure to remove the thrombus [91]. Although there are two widely used techniques available, no superiority has been established between them. The Contact Aspiration vs Stent Retriever for Successful.

Revascularization (ASTER) trial was a randomized, blinded superiority trial that investigated possible differences in reperfusion between aspiration devices and stent retrievers. Even though the reperfusion rate in direct aspiration was slightly higher (85.4%) than in stent retrieval (83.1%), the difference is not significant so no superiority could be established between the two techniques [92]. Furthermore, a non-inferiority study called the COMPASS trial was conducted in North America exploring the same question. Like the ASTER trial, direct aspiration (52%) had slightly higher good functional outcomes at 3 months compared to stent retrieval. However, no significant differences could be found between the two groups; thus, these findings indicate that direct aspiration is non-inferior to stent retrievers [93]. Ultimately, the decision to use either stent retrieval or direct aspiration is based on each individual patient and the characteristics of the clot [94].

Recently, many stroke centers have begun using a combination of a stent retriever and aspiration devices with a balloon-guided catheter to treat patients with acute ischemic stroke. This combination has resulted in high rates of reperfusion, first-pass recanalization rates, lower rates of embolization distally, and a lower number of attempts [92]. While this may be a more expensive treatment process compared to stent retriever or aspiration individually, this cost is compensated by the improved recanalization rates and shorter procedure time which both translate to more successful patient outcomes [94].

## 7. Comparison of Therapies

Although the decision to use stent retrieval versus direct aspiration varies for each patient, there are general advantages and disadvantages associated with each type of therapy. As it pertains to stent retrieval specifically, one possible complication associated with this process is severe vascular damage with associated intimal thickening [95]. Moreover, the usage of stents can cause clot fragmentation which may lead possible distal embolization and occlusion of small vessels that were previously uninvolved [96]. However, some studies have found that direct aspiration can lead to distal embolization and arterial occlusion as well [97]. Additionally, studies have also demonstrated that stent retrieval is more effective at capturing large or more dense clots that might be challenging for simple aspiration [98]. On the other hand, direct aspiration is typically used more for smaller clots, has been shown to have faster reperfusion times compared to stent retrieval, and is also considered a more cost-effective option [99,100]. Ultimately, many studies have found that the most effective treatment is a combination of stent retrieval and direct aspiration [101–103]. Thrombolytics is a non-invasive treatment option that also been very effective in the treatment of acute ischemic stroke. However, some disadvantages are that it must applied in a very limited time window (<4.5 hours since symptoms onset) and has risk of bleeding complications [104].

## 8. Conclusion

Ultimately, acute ischemic stroke is a prominent cause of death across the globe. Currently, endovascular therapy and intravenous thrombolysis are two possible treatment modalities available. Specifically, endovascular therapy has been established as the gold standard of care for acute ischemic stroke patients due to its numerous advantages of thrombolysis, such as an extended time window and fewer contraindications.

Additionally, it is associated with higher recanalization rates and good functional independent outcomes. Stent retrievers and direct aspiration are two types of mechanical thrombectomy techniques; however, studies have shown that one technique is not significantly better than the other. A combination of both stent retrievers and aspiration devices has resulted in higher rates of reperfusion and recanalization rates. Thus, technological advances in the treatment of acute ischemic stroke continue to be made which has significantly improved functional outcomes in patients.
